# Inpatient hospital performance is associated with post-discharge sepsis mortality

**DOI:** 10.1186/s13054-020-03341-3

**Published:** 2020-10-27

**Authors:** Nicholas M. Mohr, Alexis M. Zebrowski, David F. Gaieski, David G. Buckler, Brendan G. Carr

**Affiliations:** 1 Department of Emergency Medicine, University of Iowa Carver College of Medicine, 200 Hawkins Drive, 1008 RCP, Iowa City, IA 52242 USA; 2 Division of Critical Care, Department of Anesthesia, University of Iowa Carver College of Medicine, Iowa City, IA USA; 3 Department of Emergency Medicine, Thomas Jefferson University, Philadelphia, PA USA; 4 Mount Sinai University, New York, NY USA

**Keywords:** Sepsis, Quality of health care, Patient discharge, Patient readmission

## Abstract

**Background:**

Post-discharge deaths are common in patients hospitalized for sepsis, but the drivers of post-discharge deaths are unclear. The objective of this study was to test the hypothesis that hospitals with high risk-adjusted inpatient sepsis mortality also have high post-discharge mortality, readmissions, and discharge to nursing homes.

**Methods:**

Retrospective cohort study of age-qualifying Medicare beneficiaries with sepsis hospitalization between January 2013 and December 2014. Hospital survivors were followed for 180-days post-discharge, and mortality, readmissions, and new admission to skilled nursing facility were measured. Inpatient hospital-specific sepsis risk-adjusted mortality ratio (observed: expected) was the primary exposure.

**Results:**

A total of 830,721 patients in the cohort were hospitalized for sepsis, with inpatient mortality of 20% and 90-day mortality of 48%. Higher hospital-specific sepsis risk-adjusted mortality was associated with increased 90-day post-discharge mortality (aOR 1.03 per each 0.1 increase in hospital inpatient O:E ratio, 95% CI 1.03–1.04). Higher inpatient risk adjusted mortality was also associated with increased probability of being discharged to a nursing facility (aOR 1.03, 95% CI 1.02–1.03) and 90-day readmissions (aOR 1.03, 95% CI 1.02–1.03).

**Conclusions:**

Hospitals with the highest risk-adjusted sepsis inpatient mortality also have higher post-discharge mortality and increased readmissions, suggesting that post-discharge complications are a modifiable risk that may be affected during inpatient care. Future work will seek to elucidate inpatient and healthcare practices that can reduce sepsis post-discharge complications.

## Background

Sepsis is a life-threatening condition that affects 1.7 million Americans annually, with an in-hospital mortality of 16% [1]. Costing over $60 billion each year, sepsis is the most expensive acute medical condition for which Medicare reimburses hospitals [2]. Early treatment has been shown to improve survival to hospital discharge [1, 3, 4], but sepsis case-fatality continues to vary significantly by hospital (41% for highest-mortality decile of hospitals vs. 29% for lowest-mortality decile, *p* < 0.001) [5].

The burden of sepsis continues beyond hospital discharge. Many sepsis survivors have persistent neurocognitive deficits, neuromuscular weakness, symptoms of depression and post-traumatic stress disorder, and poor quality of life [6–10]. Readmissions after discharge are common (21% within 30 days) [11], and sepsis survivors have increased post-discharge mortality [12–14]. Post-discharge deaths have been associated with premorbid health status and hospital factors [15, 16], but it is unclear whether these factors are modifiable, and whether hospital care can alter the post-discharge course.

**Fig. 1:**
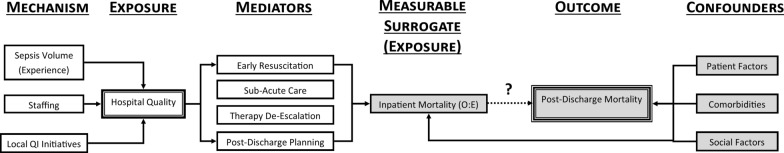
Proposed causal diagram for the hypothesized relationship between hospital-specific observed:expected (O:E) mortality ratio and post-discharge mortality. Shaded boxes indicate parameters that are measurable (non-shaded boxes are unmeasured). In our primary analysis, we are using inpatient O:E mortality as a surrogate approximation of hospital quality. The purpose of this analysis is to understand to what degree post-discharge mortality may be modifiable based on hospital-level care

The objective of this study was to determine the relationship between quality of hospital care (measured by hospital-specific inpatient adjusted sepsis survival) and post-discharge outcomes, including mortality, readmissions, and skilled nursing facility discharge (Fig. 1). We postulate that hospitals that provide higher quality care have improved sepsis risk-adjusted inpatient mortality and that this quality leads to improved post-discharge mortality in those who survive hospitalization. This analysis differs from prior work, in that we are evaluating a primary exposure of hospital performance (a more direct measure of hospital quality) rather than administrative or epidemiologic factors than may be related with quality indirectly, and our outcome includes only those who survive hospitalization [15–18]. Such a relationship would suggest that the impact of inpatient sepsis therapies may extend beyond hospital discharge, and that early treatment elements may change long-term immunity, organ function, and debility.

## Methods

### Study design, setting, and participants

This analysis was a retrospective cohort study of age-qualifying Medicare beneficiaries with an emergency department (ED)-based hospitalization in a U.S. hospital for sepsis or septic shock between January 1, 2013 and December 31, 2014. Hospital encounters were identified using the Outpatient and Medicare Provider Analysis and Review (MedPAR) research identifiable files from the Centers for Medicare and Medicaid Services (CMS) administrative claims data, while patient demographic information and dates of death were obtained from the Medicare Beneficiary Summary File and Vital Status records. All cases were followed for 6 months after hospital admission, with deaths from any cause captured through June 30, 2015 for the last-enrolling patients, and readmissions for any cause censored on December 31, 2014 due to the years of Outpatient and MedPAR data available. In-hospital mortality was estimated using data from January 1, 2013 through December 31, 2014, and these mortality estimates were used for calculating observed-to-expected (O:E) mortality ratios for inclusion in subsequent explanatory models. Only hospitals with at least 200 cases during the 2-year period were included in the analysis to allow for more stable O:E ratio estimates. This study was approved by the local institutional review board (IRB) and is reported using the STrengthening the Reporting of OBservational studies in Epidemiology (STROBE) statement [20].

### Definitions

*Severe sepsis or septic shock* was defined according to *International Classification of Diseases, 9th edition, Clinical Modification* (ICD-9-CM) diagnosis codes for septicemia with an additional diagnosis for organ dysfunction, as previously reported [21–23]. *Rural* residence was defined based on the county of residence, and was classified according to the 2013 Rural–Urban Continuum Codes (RUCC) published by the U.S. Department of Agriculture [24]. *Comorbidities* were defined using the Elixhauser method, which identifies a set of 30 comorbid conditions from administrative data associated with increased mortality, length-of-stay, and charges [25]. *Index hospital* was the first hospital where a patient was seen for a given sepsis episode, while *final hospital* was the last hospital where a patient was seen (to account for patients transferred between hospitals for their care).

### Exposures

#### Hospital-specific mortality

The primary exposure was hospital-specific sepsis mortality. Hospital-specific sepsis mortality was reported as an O:E mortality ratio based strictly on in-hospital mortality for the final hospital in which a patient was treated, calculated for the study period 2013–2014. A multivariable logistic regression model was constructed with an outcome of in-hospital mortality and a priori*-*defined patient-level predictors, including age, race, sex, comorbidities, infection source, organ dysfunction, skilled nursing facility residence prior to admission, community factors (percent Black, percent Hispanic, percent with high school degree or higher, percent below poverty line), ICU services in hospital, and teaching hospital. All variables were selected based on theory and the proposed relationship with exposures and outcomes, and the same adjustment variables were used for all models [5, 17, 26]. All continuous variables were modeled in categories, and interactions were tested. The predicted probability of mortality was generated for each case in the data set, then the sum of observed in-hospital mortality and predicted in-hospital mortality for each facility was calculated. The ratio of the observed mortality to the predicted mortality was the O:E ratio and was the primary predictor in this analysis.

### Primary and secondary outcomes

#### Primary outcome

The primary outcome was 90-day post-discharge mortality, measured from the date of hospital discharge among patients who survived their initial sepsis hospitalization. The primary analysis used a hierarchical logistic regression model clustered on the final hospital with the primary exposure of hospital-specific O:E mortality ratio and other potential patient and system-oriented confounders. Interactions were tested and all continuous variables were coded into categorical groups. Covariates for the model were selected based on prior data and possible confounders in patient-level, facility-level, and community-level variables.

#### Secondary analyses

Secondary analytic models were developed to measure mortality at 30 days, 60 days, and 180 days post-discharge. A sensitivity analysis was performed using the hospital O:E ratio for the index hospital, rather than the final hospital, to determine if assigning ownership to the first hospital changed the association with post-discharge outcomes. Multivariable models were built similarly to measure the association between hospital-specific predictors on hospital discharge to skilled nursing facility (only among cases that did not reside in a skilled nursing facility at the time of index admission) and readmission within the first 90-days after discharge.

### Sensitivity analysis

#### Survival models

An alternative approach was planned a priori to function as a sensitivity analysis (if the impact of hospital mortality on post-discharge mortality appeared preserved across time, supporting the proportional hazards assumption): a Cox proportional hazards model was developed with the same set of covariates and an outcome of time-to-death, among patients who survived the initial hospitalization. A second proportional hazards model was built for time-to-readmission among hospital survivors.

#### Unobserved confounder

An “*E*-value” was calculated to determine the minimum strength of association required with both the predictor and the outcome, for an unobserved confounder to render the coefficient of our primary exposure null. In this way, we estimated how significant an unobserved variable would have to be for our results to be attributable to confounding bias alone. To interpret this value, the reader might conclude that a confounding variable not included in the model would have to have an odds ratio of at least this value for our findings to be a result of confounding alone.

### Analysis

For all multivariable models, continuous predictor variables were categorized to avoid the linearity assumption, interactions were tested for significance, and variables were screened for multicollinearity. All analyses are done using complete case analysis, and goodness of fit was assessed using area under the curve (AUC) of the final model. All statistical tests were considered significant for *p* < 0.05 using two-tailed tests, and all analysis was conducted using Stata v.15.1. (StataCorp LLC, College Station, Texas).

## Results

**Fig. 2:**
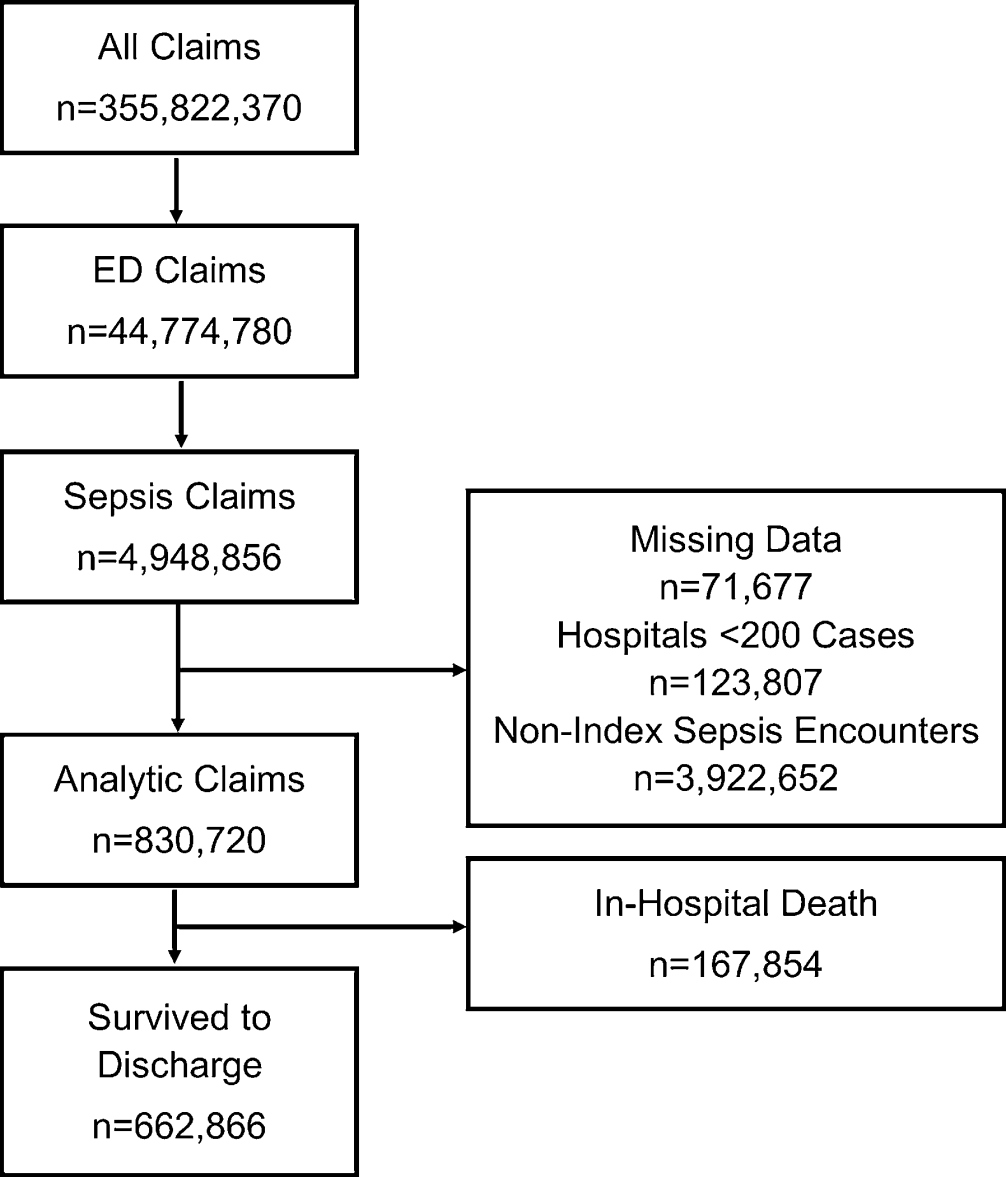
Flow diagram of study subjects. Non-index sepsis encounters include prior admissions, readmissions, transfers (at the receiving hospital), and skilled nursing facility admissions

A total of 830,720 age-qualifying Medicare beneficiaries were hospitalized for sepsis between 2013 and 2014 (Fig. 2). We excluded 123,807 admissions in 1922 low-volume hospitals with fewer than 200 cases during the sample period. Most patients (68%) were hospitalized with either a respiratory or urinary infection source, 14% had a co-existing malignancy, and 9% were admitted from a skilled nursing facility (Table 1). Of all cases, 1% were transferred between hospitals. In-hospital mortality was 20% (*n* = 167,854), with 30-day, 90-day, and 180-day cumulative mortality of 35% (*n* = 291,708), 43% (*n* = 353,147), and 48% (*n* = 398,914), respectfully. Of sepsis discharges, 37% (*n* = 247,045) were readmitted to a hospital within 180 days, with the median time to readmission approximately 32 days (IQR 12–73 days).

**Table 1:** Baseline characteristics of the study population

Characteristic	Descriptive statistics	
	All cases (*n* = 830,720)	Survived to discharge (*n* = 662,866)
	*n* (%)	*n* (%)
Patient characteristics		
Sex		
Male	393,852 (47.4)	314,824 (47.5)
Female	436,868 (52.6)	348,042 (52.5)
Race		
White	641,526 (77.2)	512,243 (77.3)
Black	101,581 (12.2)	79,308 (12.0)
Other	87,613 (10.6)	71,315 (10.8)
Age		
65–74 years	274,021 (33.0)	225,032 (33.9)
75–84 years	303,933 (36.6)	242,410 (36.6)
85+ years	252,766 (30.4)	195,424 (29.5)
Urbanicity of residence (by RUCC code)		
Urban (metro counties)	740,872 (89.2)	590,784 (89.1)
Rural (nonmetro counties)	89,377 (10.8)	71,715 (10.8)
Other/unknown	471 (0.1)	367 (0.1)
Cancer diagnosis		
None	715,653 (86.2)	580,536 (87.6)
Metastatic solid tumor	10,656 (1.3)	7,208 (1.1)
Non-metastatic solid tumor	72,133 (8.7)	51,807 (7.8)
Hematologic malignancy	32,278 (3.9)	23,315 (3.5)
Infection source		
Urinary tract	338,046 (40.7)	288,389 (43.5)
Pneumonia	225,647 (27.2)	171,908 (25.9)
Abdominal	33,433 (4.0)	24,135 (3.6)
Bloodstream	26,217 (3.2)	22,148 (3.3)
Cellulitis	24,288 (2.9)	21,407 (3.2)
Bone	17,909 (2.2)	15,247 (2.3)
Surgical	10,960 (1.3)	9329 (1.4)
Ear, nose, throat	1240 (0.2)	1130 (0.2)
Meningitis	1051 (0.1)	832 (0.1)
Gastrointestinal	794 (0.1)	755 (0.1)
Other/unknown	151,135 (18.2)	107,586 (16.2)
Organ dysfunction		
Renal	250,655 (30.2)	212,506 (32.1)
Neurologic	227,220 (27.4)	177,610 (26.8)
Metabolic	145,086 (17.5)	101,214 (15.3)
Hematologic	73,996 (8.9)	61,618 (9.3)
Cardiac	50,414 (6.1)	39,498 (6.0)
Respiratory	41,985 (5.1)	35,072 (5.3)
Hepatic	8396 (1.0)	5525 (0.8)
Other/unknown	32,968 (4.0)	29,823 (4.5)
Admission from skilled nursing facility		
No	759,098 (91.4)	618,575 (93.3)
Yes	71,622 (8.6)	50,291 (7.6)
Hospital characteristics		
ICU services available		
No	10,409 (1.3)	8,633 (1.3)
Yes	736,934 (88.7)	588,136 (88.7)
Not reported/unknown	83,377 (10.0)	66,097 (10.0)
Council of teaching hospitals member		
No	675,249 (81.3)	541,556 (81.7)
Yes	150,440 (18.1)	117,176 (17.7)
Not reported/unknown	5031 (0.6)	4134 (0.6)
Community characteristics (zip code)		
Percent of population unemployed		
0–5%	95,725 (11.5)	77,264 (11.7)
6–8%	260,369 (31.3)	208,787 (31.5)
9–11%	230,791 (27.8)	184,317 (27.8)
12–15%	155,421 (18.7)	123,043 (18.6)
≥ 16%	88,414 (10.6)	69,455 (10.5)
Percent of population, black or African American		
0–1%	183,904 (22.1)	148,852 (22.5)
2–3%	145,025 (17.5)	116,636 (17.6)
4–5%	87,997 (10.6)	70,587 (10.6)
6–10%	124,661 (15.0)	99,529 (15.0)
11–20%	114,407 (13.8)	90,978 (13.7)
21–40%	90,602 (10.9)	71,360 (10.8)
≥ 41%	84,124 (10.1)	64,924 (9.8)
Percent of population, Hispanic		
0–1%	90,385 (10.9)	72,473 (10.9)
2–3%	153,434 (18.5)	123,561 (18.6)
4–5%	106,717 (12.9)	85,911 (13.0)
6–10%	156,509 (18.8)	124,846 (18.8)
11–20%	136,061 (16.4)	108,231 (16.3)
21–40%	101,608 (12.2)	803,78 (12.1)
≥ 41%	86,006 (10.4)	67,466 (10.2)
Percent of population, high school degree or higher		
0–20%	105,685 (12.7)	83,642 (12.6)
21–25%	129,294 (15.6)	102,210 (15.4)
26–28%	103,013 (12.4)	82,093 (12.4)
29–33%	177,288 (21.3)	142,046 (21.4)
34–38%	149,051 (17.9)	119,137 (18.0)
39–45%	107,329 (12.9)	86,421 (13.0)
≥ 46%	59,060 (7.1)	47,317 (7.1)
Percent of population, below poverty line		
0–7%	159,421 (19.2)	128,113 (19.3)
8–10%	118,812 (14.3)	95,364 (14.4)
11–15%	183,827 (22.1)	147,216 (22.2)
16–18%	91,917 (11.1)	73,484 (11.1)
19–25%	152,570 (18.4)	121,147 (18.3)
≥ 26%	124,173 (15.0)	97,542 (14.7)

### Association between in-hospital mortality and post-discharge mortality

**Fig. 3:**
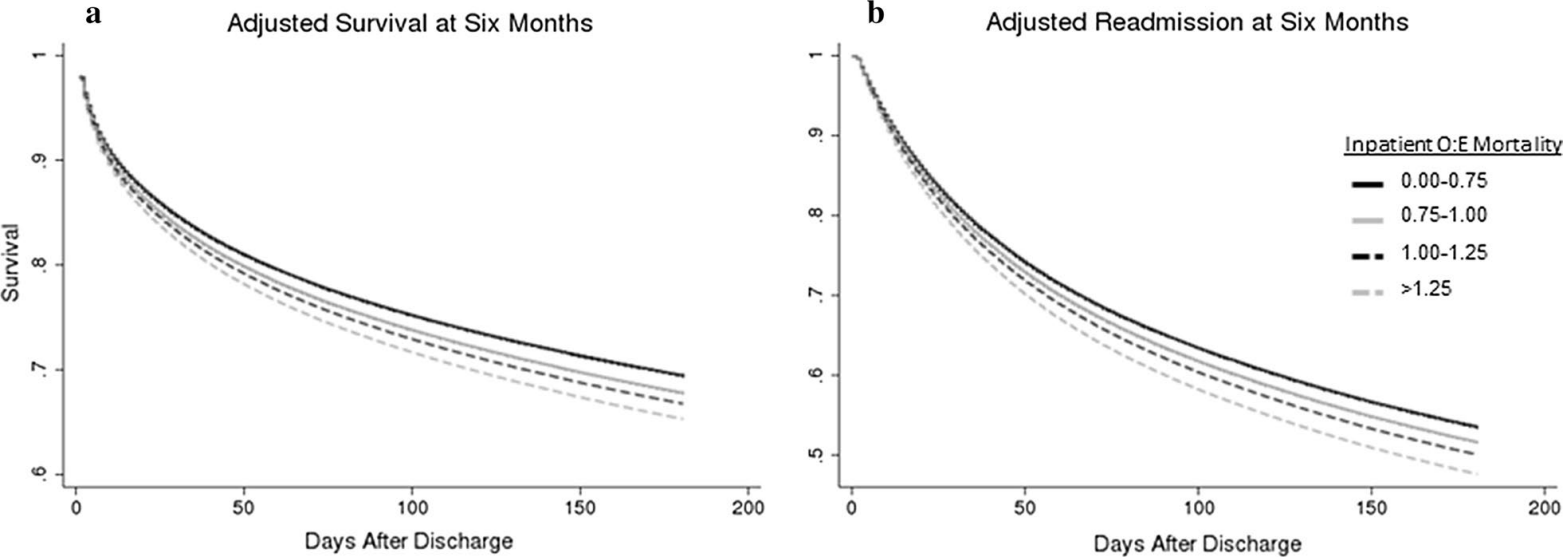
Cox proportional hazard model curves showing adjusted time-to mortality (**a**) and time-to-readmission (**b**) for patients who survive a sepsis hospitalization. Curves are stratified into cohorts defined by the quartile of observed:expected (O:E) in-hospital sepsis mortality aggregated at the level of the hospital. Survival analysis is adjusted for age, race, sex, comorbidities, infection source, organ dysfunction, skilled nursing facility residence prior to admission, community factors (percent Black, percent Hispanic, percent with high school degree or higher, percent below poverty line), ICU services in hospital, teaching hospital

The median index hospital O:E ratio was 1.00 (IQR 0.83–1.17), with 1585 hospitals reporting at least 200 cases over the 2-year period. O:E ratios ranged from 0.29 to 2.20 (IQR 0.83–1.17, Additional file 1: Fig. S1, Additional file 2: Table S1). In univariable modeling, increasing hospital-specific O:E ratios were associated with increased post-discharge mortality at 90-days (unadjusted OR 1.08 for each 0.1 increase in hospital-specific O:E ratio, 95% CI 1.08–1.08). Controlling for potentially confounding patient-, facility-, and community-level covariates, the adjusted odds ratio showed a strong association between hospital O:E ratios and mortality (aOR 1.03 for each 0.1 increase in hospital-specific O:E ratio, 95% CI 1.03–1.04). The magnitude of effect was unchanged for mortality from 30 to 180 days (Fig. 3, Additional file 3: Table S2).

### Secondary outcomes

No differences were seen in the association between index hospital O:E and 90-day post-discharge mortality compared with the use of final hospital in the primary models (aOR 1.03, 1.03–1.04). Of the 610,874 patients that did not live in a nursing facility prior to their index sepsis admission and who survived to discharge, 25% (*n* = 153,378) were discharge to a SNF. Using a multivariable model similar to the primary model, patients from hospitals with the highest O:E ratios had an increased odds of being discharged to a skilled nursing facility (aOR 1.03 per 0.1 increase in O:E mortality ratio for final hospital, 95% CI 1.02–1.03). Similarly, patients from hospitals with the highest risk-adjusted mortality also had increased probability of 90-day readmissions (aOR 1.03 per 0.1 increase in O:E for final hospital, 95% CI 1.02–1.03). By 180-days after discharge, the adjusted odds of readmission had decreased to 1.02 per 0.1 increase in O:E (95% CI 1.02–1.03).

### Survival analysis

As a sensitivity analysis, a Cox proportional hazards model was used to measure the time to death, accounting for all the covariates from the logistic regression models and the hospital O:E ratio (Fig. 3). The hazard ratio for post-discharge mortality was higher in patients treated in a hospital with a higher O:E ratio (aHR 1.02 per 0.1 increase in O:E for final hospital, 95% CI 1.02–1.03). A “dose–response” relationship was seen between hospital O:E and mortality with lower hazard for post-discharge mortality in the lower categories of hospital O:E (reference group < 0.75; 0.75–1.00: aHR 1.01, 95% CI 1.00–1.01; 1.00–1.25: aHR 1.01, 95% CI 1.01–1.01; > 1.25: aHR 1.02, 95% CI 1.01–1.02). A Cox proportional hazards model was also used to assess time to readmission. In adjusted models, higher hospital O:E ratios were associated with increased readmissions (aHR: 1.02 per 0.1 increase in O:E for index hospital, 95% CI 1.02–1.03). For hospitals with an O:E ratio > 1.25, median time to readmission within the first 180 days after discharge was 30 days (IQR 11–69) compared with 34 days in hospitals with an O:E < 0.75 (IQR 13–76).

In our sensitivity analysis to measure the theoretical magnitude of an unobserved confounder (*E*-value), the magnitude of bias introduced by an unmeasured variable must have an odds of at least 2.05 (lower 95% CI 1.88). A moderately strong predictor of both hospital O:E and death at 90 days would be required to result in null findings (similar in magnitude to premorbid cancer diagnosis or nursing home residence). It seems unlikely that an unobserved variable predicting mortality with a magnitude equal to nursing home residence exists.

## Discussion

The relationship between sepsis and post-discharge mortality has been well established [12–14, 27], but whether post-discharge mortality has modifiable treatment-related risks remained unclear. This question has been challenging to answer, since the specific biological mechanisms by which post-sepsis mortality is elevated have not been well elucidated. Mechanistic data suggest that sepsis survivors remain immune suppressed [28, 29], have increased rates of atherosclerosis [13, 30], and that epigenetic regulation may play a role in modulating these effects [31]. The question that mechanistic studies have not answered, however, is whether improved inpatient care can attenuate the impact on late post-discharge mortality. By analyzing variation between U.S. hospitals, our data suggests that it may.

Inpatient quality can be difficult to measure [32, 33], and the factors associated with sepsis quality are myriad [34, 35]. The Early Management Bundle for Severe Sepsis/Septic Shock (SEP-1) [19] reported by the Centers for Medicare and Medicaid Services is one parameter for ranking hospitals on adherence with a single bundle of very early care, but this measure is not a comprehensive measure of sepsis quality of care and the specific causal relationship between SEP-1 performance and outcomes has been questioned [36–40]. By using an inclusive measure of risk-adjusted inpatient sepsis mortality aggregated at the facility level, we have attempted to capture quality-of-care by its outcome. This study design cannot identify *which* factors are associated with preventing late mortality or readmissions, but it provides evidence that care factors that affect inpatient outcomes may continue to influence patients after hospital discharge.

What factors might be responsible for the magnitude of the association? Factors associated with post-discharge death could fall into one of four categories: early resuscitation interventions, sub-acute organ support and infection management, de-escalation of therapy, and post-discharge planning and care coordination. Early care interventions, such as timely antibiotics and hemodynamic resuscitation could decrease organ failure and limit the degree of subclinical organ dysfunction persisting at hospital discharge. Immunomodulatory effects of resuscitation may prevent both early organ failure and persistent immune dysfunction [43].

The overall elements of care that are performed after initial resuscitation, including antibiotic use and discontinuation, diuresis, early mobility, delirium management, nutrition, and other factors may also contribute to sepsis outcomes [4, 44–46], but the specific manner in which these practices function as predictors and effect modifiers is not well described. Early mobility, timely extubation, and delirium prevention strategies are well-described methods of decreasing mortality and disability from critical illness, and thus are attractive targets to investigate in future studies [47–49]. Finally, discharge planning and care coordination may play a significant role in post-discharge follow-up, access to care, medication management, and the transition to community living [50]. Discharge planning, care coordination, and degree of pre-discharge medical recovery also may explain the relationship between inpatient O:E mortality, new skilled nursing discharges, and readmissions, but none of these elements were tested in our analysis. Gadre, et al. recently reported readmissions after nearly 18% of sepsis discharges, and they were associated with more comorbidities, longer index hospital stays, and residence in skilled nursing facilities [51]. Although conclusive causal relationships have not been established, one might predict that (1) providing high-quality sepsis care that limits deconditioning and chronic organ dysfunction, (2) scheduling discharge timing and managing chronic disease well, and (3) developing strong relationships with primary care providers, community organizations, and post-acute care providers to provide a seamless post-acute care transition may effectively limit the need for both skilled nursing and readmissions.

This study has several limitations. First, our use of observational data limits our statistical models to variables commonly recorded in administrative data, allowing for the possibility of unobserved confounders. These available data also led to our use of hospital O:E mortality as a surrogate for hospital quality—likely an incomplete surrogate with some potential for residual confounding and sensitive to differences in coding practices between hospitals. Second, we included only Medicare beneficiaries in these data. While two-thirds of sepsis cases in the U.S. are Medicare beneficiaries, using these cases alone isolates a population over age 65 with reliable payment for healthcare services.

## Conclusions

In conclusion, hospitals with the lowest risk-adjusted sepsis inpatient mortality also have reduced post-discharge mortality. These observations suggest that some factors influencing in-hospital mortality also influence post-discharge outcomes, and some of these factors may be modifiable. Future work should focus on identifying factors associated with post-discharge outcomes and optimizing inpatient and transitional care to improve the likelihood of functional long-term recovery.

## Supplementary information

**Additional file 1: Figure S1**. Distribution of Hospital Sepsis Observed: Expected Mortality Ratio for Included Facilities.

**Additional file 2: Table S1**. Hospital characteristics by observed to expected (O:E) ratio of in-hospital mortality

**Additional file 3: Table S2**. Outcomes

## Data Availability

Because of confidentiality restrictions with the data sets used, the data set used for this analysis is not available for sharing.

## Abbreviations

aHR: Adjusted hazard ratio
aOR: Adjusted odds ratio
CMS: Centers for Medicare and Medicaid Services
ED: Emergency department
ICD-9-CM: *International Classification of Diseases, 9th edition, Clinical Modification*
IRB: Institutional review board
IQR: Interquartile range
MedPAR: Medicare Provider Analysis and Review
O:E: Observed-to-expected
OR: Odds ratio
RUCC: Rural-Urban Continuum Codes
SEP-1: Early Management Bundle for Severe Sepsis/Septic Shock
STROBE: STrengthening the Reporting of OBservational studies in Epidemiology
95% CI: 95-Percent confidence interval
