# Supplementary figures and images for: Inpatient hospital performance is associated with post-discharge sepsis mortality
Source: Crit Care. 2020 Oct 27;24:626. doi: 10.1186/s13054-020-03341-3 (PMC7592563; doi:10.1186/s13054-020-03341-3)

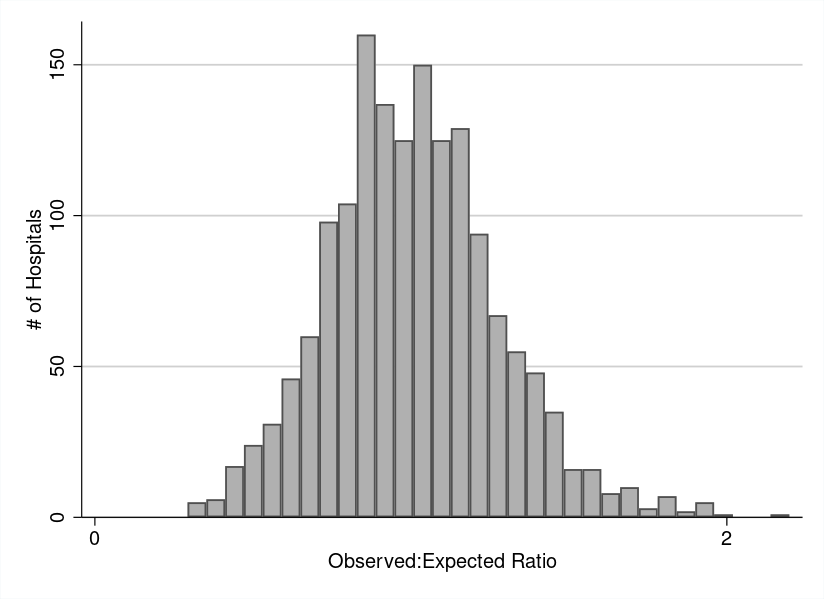

Supplement: Supplementary file 1 — Additional file 1: Figure S1. Distribution of Hospital Sepsis Observed: Expected Mortality Ratio for Included Facilities. [file 13054_2020_3341_MOESM1_ESM.png]
