# Supplementary material for: Inpatient hospital performance is associated with post-discharge sepsis mortality
Source: Crit Care. 2020 Oct 27;24:626. doi: 10.1186/s13054-020-03341-3 (PMC7592563; doi:10.1186/s13054-020-03341-3)
Supplement: Supplementary file 2 — Additional file 2: Table S1. Hospital characteristics by observed to expected (O:E) ratio of in-hospital mortality [file 13054_2020_3341_MOESM2_ESM.docx]

**Table S1.** Hospital characteristics by observed to expected (O:E) ratio of in-hospital mortality

| **Characteristic** | **O:E** | | | |
| --- | --- | --- | --- | --- |
|  | 0.00-0.75 | 0.75-1.00 | 1.00-1.25 | >1.25 |
|  | *n (%)* | *n (%)* | *n (%)* | *n (%)* |
| Hospitals | 261 | 545 | 516 | 263 |
| Cases | 143,088 | 293,217 | 261,866 | 132,549 |
| Disposition |  |  |  |  |
| Home | 56,942 (41.1) | 97,658 (34.8) | 77,298 (31.1) | 34,451 (27.1) |
| Rehab | 3,858 (2.8) | 8,308 (3.0) | 7,280 (2.9) | 3,207 (2.5) |
| Died/Hospice | 31,102 (22.4) | 78,102 (27.8) | 78,293 (31.5) | 46,924 (36.9) |
| SNF/LTCH | 45,252 (32.7) | 94,104 (33.5) | 83,205 (33.5) | 41,258 (32.5) |
| Transfer | 1,425 (1.0) | 2,794 (1.0) | 2,651 (1.1) | 1,232 (1.0) |
| Length of Stay median (IQR) | 5.0 (3.0, 9.0) | 6.0 (4.0, 10.0) | 7.0 (4.0, 12.0) | 8.0 (4.0, 14.0) |
| Transfer to SNF | 32,567 (22.8) | 67,375 (23.0) | 57,833 (22.1) | 27,791 (21.0) |
| ICU Services Available |  |  |  |  |
| No | 8 (3.1) | 8 (1.5) | 6 (1.2) | 1 (0.4) |
| Yes | 230 (88.1) | 479 (87.9) | 443 (85.9) | 221 (84.0) |
| Not Reported | 23 (8.8) | 58 (10.6) | 67 (13.0) | 41 (15.6) |
| Annual Admissions, median (IQR) | 9,608  (6,039, 16,060) | 12,630  (8,515, 19,850) | 14,216  (9,543, 21,587) | 14,323  (9,561, 20,628) |
| Sepsis volume, Quintiles |  |  |  |  |
| 1st | 11,695 (8.4) | 18,393 (6.5) | 18,886 (7.6) | 15,581 (12.3) |
| 2nd | 31,507 (22.7) | 60,307 (21.5) | 54,800 (22.0) | 32,770 (25.8) |
| 3rd | 28,018 (20.3) | 61,036 (21.7) | 62,908 (25.3) | 28,563 (22.5) |
| 4th | 32,012 (23.1) | 64,246 (22.9) | 67,824 (27.3) | 20,399 (16.1) |
| 5th | 35,300 (25.5) | 76,902 (27.4) | 44,186 (17.8) | 29,712 (23.4) |
| Teaching Hospital |  |  |  |  |
| No | 240 (92.0%) | 466 (85.5%) | 444 (86.0%) | 226 (85.9%) |
| Yes | 19 (7.3%) | 75 (13.8%) | 70 (13.6%) | 37 (14.1%) |
| Not reported/ Unknown | 2 (0.8%) | 4 (0.7%) | 2 (0.4%) | 0 (0.0%) |
