# Supplementary material for: Inpatient hospital performance is associated with post-discharge sepsis mortality
Source: Crit Care. 2020 Oct 27;24:626. doi: 10.1186/s13054-020-03341-3 (PMC7592563; doi:10.1186/s13054-020-03341-3)
Supplement: Supplementary file 3 — Additional file 3: Table S2. Outcomes [file 13054_2020_3341_MOESM3_ESM.docx]

**Table S2. Outcomes.**

| Variable | 30 Day Mortality  OR (95% CI) | 60 Day Mortality OR (95% CI) | 90 Day Mortality  OR (95% CI) | 180 Day Mortality OR (95% CI) |
| --- | --- | --- | --- | --- |
| Final Index Hospital O:E Ratio (per 0.1 unit increase) | 1.02 (1.01, 1.02)* | 1.03 (1.03, 1.04)* | 1.03 (1.03, 1.04)* | 1.03 (1.03, 1.04)* |
| Age Category *(Ref. 65-74 yrs)* |  |  |  |  |
| 75-84 yrs | 1.46 (1.43, 1.48)* | 1.28 (1.24, 1.31)* | 1.27 (1.23, 1.31)* | 1.22 (1.20, 1.25)* |
| ≥85 yrs | 2.39 (2.35, 2.43)* | 1.81 (1.76, 1.87)* | 1.76 (1.69, 1.82)* | 1.75 (1.71, 1.79)* |
| Race *(Ref. White)* |  |  |  |  |
| Black | 0.96 (0.93, 0.99)* | 1.17 (1.13, 1.22)* | 1.22 (1.16, 1.28)* | 1.24 (1.20, 1.29)* |
| Other/Unknown | 0.83 (0.81, 0.86)* | 0.94 (0.91, 0.98)* | 0.95 (0.92, 1.00)* | 0.94 (0.91, 0.97)* |
| Sex *(Ref Male)* |  |  |  |  |
| Female | 1.01 (1.00, 1.02) | 0.95 (0.93, 0.98)* | 0.94 (0.92, 0.97)* | 0.92 (0.90, 0.93)* |
| Elixhauser Comorbidities |  |  |  |  |
| Congestive Heart Failure | 1.34 (1.32, 1.36)* | 1.35 (1.32, 1.39)* | 1.36 (1.32, 1.40)* | 1.37 (1.34, 1.40)* |
| Cardiac Arrhythmias | 1.19 (1.17, 1.21)* | 1.19 (1.16, 1.21)* | 1.19 (1.15, 1.22)* | 1.17 (1.14, 1.19)* |
| Valvular Disease | 0.94 (0.92, 0.96)* | 1.03 (0.99, 1.06) | 0.98 (0.94, 1.02) | 1.03 (1.00, 1.07)* |
| Diabetes,Complicated | 0.85 (0.83, 0.87)* | 0.96 (0.92, 1.00)* | 1.02 (0.97, 1.07) | 1.04 (1.00, 1.08)* |
| Renal Failure | 1.09 (1.07, 1.10)* | 1.19 (1.16, 1.22)* | 1.19 (1.16, 1.23)* | 1.24 (1.21, 1.27)* |
| Liver Disease | 1.59 (1.55, 1.64)* | 1.29 (1.23, 1.35)* | 1.24 (1.17, 1.32)* | 1.20 (1.15, 1.26)* |
| Chronic Pulmonary Disease | 0.99 (0.97, 1.00)* | 1.10 (1.08, 1.13)* | 1.09 (1.06, 1.12)* | 1.15 (1.12, 1.17)* |
| Other Neurological Disorders | 1.29 (1.26, 1.32)* | 1.22 (1.18, 1.26)* | 1.21 (1.15, 1.27)* | 1.25 (1.21, 1.29)* |
| Diabetes, Uncomplicated | 0.87 (0.86, 0.88)* | 0.92 (0.89, 0.94)* | 0.96 (0.93, 0.99)* | 0.99 (0.97, 1.02) |
| Obesity | 0.64 (0.62, 0.65)* | 0.70 (0.67, 0.73)* | 0.75 (0.72, 0.79)* | 0.73 (0.71, 0.76)* |
| Alcohol Abuse | 1.46 (1.31, 1.62)* | 1.30 (1.09, 1.54)* | 1.35 (1.08, 1.69)* | 1.22 (1.03, 1.45)* |
| Drug Abuse | 0.47 (0.24, 0.94)* | 0.94 (0.50, 1.77) | 0.31 (0.08, 1.26) | 0.77 (0.40, 1.47) |
| Urbanicity *(Ref. Urban)* |  |  |  |  |
| Rural | 0.99 (0.95, 1.03) | 1.04 (0.99, 1.08) | 0.99 (0.95, 1.04) | 0.99 (0.95, 1.02) |
| Other/Unknown | 1.10 (0.87, 1.38) | 1.05 (0.68, 1.62) | 0.48 (0.22, 1.04) | 0.99 (0.63, 1.54) |
| Cancer Diagnosis | 2.73 (2.68, 2.78)* | 2.75 (2.67, 2.83)* | 2.73 (2.64, 2.83)* | 2.87 (2.79, 2.95)* |
| Infection Source *(Ref. GI)* |  |  |  |  |
| Cellulitis | 1.03 (0.81, 1.32) | 1.20 (0.81, 1.78) | 0.93 (0.59, 1.47) | 1.14 (0.83, 1.57) |
| Meningitis | 1.25 (0.91, 1.73) | 0.99 (0.56, 1.72) | 0.72 (0.38, 1.37) | 0.62 (0.38, 1.01) |
| Bloodstream | 1.53 (1.20, 1.95)* | 1.86 (1.25, 2.74)* | 1.46 (0.93, 2.29) | 1.51 (1.10, 2.08)* |
| Ear, nose, throat | 0.84 (0.63, 1.13) | 0.69 (0.40, 1.20) | 1.01 (0.59, 1.74) | 0.63 (0.41, 0.96)* |
| Pneumonia | 1.61 (1.27, 2.05)* | 1.60 (1.08, 2.35)* | 1.20 (0.77, 1.88) | 1.30 (0.95, 1.79) |
| Abdominal | 1.47 (1.15, 1.87)* | 1.45 (0.98, 2.14) | 1.07 (0.68, 1.67) | 1.02 (0.74, 1.41) |
| Urinary tract | 1.31 (1.03, 1.67)* | 1.52 (1.03, 2.25)* | 1.20 (0.78, 1.90) | 1.33 (0.97, 1.82) |
| Bone | 1.64 (1.28, 2.09)* | 2.17 (1.46, 3.21)* | 1.56 (0.99, 2.46) | 1.68 (1.21, 2.32)* |
| Surgical | 0.98 (0.76, 1.25) | 1.09 (0.73, 1.63) | 0.91 (0.58, 1.44) | 0.95 (0.68, 1.32) |
| Other/Unknown | 1.78 (1.40, 2.26)* | 1.55 (1.05, 2.29)* | 1.18 (0.76, 1.84) | 1.24 (0.90, 1.69) |
| Organ dysfunction *(Ref. Resp.)* |  |  |  |  |
| Cardiac | 0.97 (0.93, 1.01) | 1.12 (1.05, 1.20)* | 1.05 (0.97, 1.14) | 1.09 (1.02, 1.16)* |
| Renal | 0.90 (0.87, 0.93)* | 1.00 (0.94, 1.06) | 0.96 (0.90, 1.02) | 0.96 (0.92, 1.01) |
| Hepatic | 1.05 (0.96, 1.13) | 1.15 (1.01, 1.31)* | 1.04 (0.88, 1.23) | 0.95 (0.83, 1.09) |
| Hematologic | 0.86 (0.83, 0.90)* | 0.98 (0.92, 1.05) | 0.96 (0.89, 1.04) | 0.96 (0.91, 1.02) |
| Metabolic | 1.22 (1.17, 1.26)* | 1.18 (1.11, 1.25)* | 1.07 (1.00, 1.14) | 1.05 (1.00, 1.11) |
| Neurologic | 1.18 (1.14, 1.23)* | 1.24 (1.16, 1.32)* | 1.14 (1.06, 1.23)* | 1.11 (1.05, 1.18)* |
| Other/Unknown | 0.70 (0.67, 0.74)* | 1.01 (0.94, 1.09) | 0.99 (0.91, 1.09) | 1.13 (1.06, 1.20)* |
| Transfer from SNF | 2.45 (2.39, 2.51)* | 2.23 (2.16, 2.31)* | 2.09 (2.01, 2.18)* | 1.92 (1.85, 1.99)* |
| % of population unemployed *(Ref. 0-5)* |  |  |  |  |
| 6-8% | 1.00 (0.97, 1.03) | 1.01 (0.97, 1.05) | 0.96 (0.92, 1.01) | 1.01 (0.98, 1.05) |
| 9-11% | 1.05 (1.01, 1.09)* | 1.03 (0.98, 1.08) | 0.98 (0.93, 1.03) | 1.01 (0.97, 1.05) |
| 12-15% | 1.07 (1.02, 1.12)* | 1.06 (1.01, 1.12)* | 0.96 (0.90, 1.02) | 1.03 (0.99, 1.08) |
| ≥16% | 1.11 (1.05, 1.17)* | 1.05 (0.99, 1.11) | 0.95 (0.88, 1.02) | 1.01 (0.96, 1.07) |
| % of population Black *(Ref. <2)* |  |  |  |  |
| 2-3% | 1.03 (1.00, 1.06)* | 1.00 (0.96, 1.04) | 1.01 (0.97, 1.05) | 1.02 (0.99, 1.06) |
| 4-5% | 1.09 (1.05, 1.12)* | 1.07 (1.02, 1.12)* | 1.03 (0.97, 1.08) | 1.05 (1.01, 1.09)* |
| 6-10% | 1.11 (1.08, 1.15)* | 1.09 (1.04, 1.14)* | 1.07 (1.02, 1.12)* | 1.07 (1.03, 1.11)* |
| 11-20% | 1.14 (1.10, 1.19)* | 1.11 (1.06, 1.16)* | 1.08 (1.03, 1.14)* | 1.07 (1.03, 1.11)* |
| 21-40% | 1.16 (1.12, 1.21)* | 1.10 (1.05, 1.15)* | 1.12 (1.06, 1.18)* | 1.07 (1.02, 1.11)* |
| ≥41% | 1.21 (1.15, 1.27)* | 1.19 (1.12, 1.25)* | 1.19 (1.11, 1.27)* | 1.13 (1.07, 1.19)* |
| % of population Hispanic *(<2)* |  |  |  |  |
| 2-3% | 0.99 (0.96, 1.02) | 1.00 (0.95, 1.04) | 0.95 (0.90, 1.00)* | 1.00 (0.96, 1.04) |
| 4-5% | 1.00 (0.96, 1.04) | 0.97 (0.93, 1.02) | 0.94 (0.89, 1.00)* | 0.99 (0.95, 1.03) |
| 6-10% | 0.98 (0.94, 1.02) | 0.93 (0.89, 0.98) | 0.96 (0.91, 1.01) | 0.98 (0.94, 1.02) |
| 11-20% | 0.99 (0.95, 1.03) | 1.01 (0.96, 1.06) | 0.97 (0.92, 1.03) | 0.99 (0.95, 1.04) |
| 21-40% | 1.01 (0.97, 1.06) | 1.03 (0.97, 1.08) | 1.02 (0.96, 1.09) | 1.00 (0.96, 1.06) |
| ≥41% | 1.06 (0.99, 1.12) | 1.11 (1.05, 1.18) | 1.04 (0.97, 1.12) | 1.09 (1.03, 1.15)* |
| % of population HS degree or greater *(Ref. 0-20)* |  |  |  |  |
| 21-25% | 1.03 (1.00, 1.06) | 1.00 (0.96, 1.04) | 1.03 (0.98, 1.09) | 1.02 (0.99, 1.06) |
| 26-28% | 1.04 (1.01, 1.07)* | 1.01 (0.97, 1.06) | 1.07 (1.01, 1.13)* | 1.00 (0.96, 1.04) |
| 29-33% | 1.05 (1.02, 1.08)* | 0.99 (0.96, 1.03) | 1.03 (0.98, 1.08) | 1.00 (0.96, 1.04) |
| 34-38% | 1.08 (1.05, 1.12)* | 1.02 (0.98, 1.07) | 1.06 (1.01, 1.11) | 1.02 (0.99, 1.06) |
| 39-45% | 1.09 (1.05, 1.13)* | 1.02 (0.97, 1.07) | 1.06 (1.00, 1.12) | 1.03 (0.99, 1.07) |
| ≥46% | 1.08 (1.04, 1.13)* | 1.06 (1.00, 1.12) | 1.03 (0.97, 1.11) | 1.02 (0.97, 1.07) |
| % of population below the poverty line *(Ref. 0-7)* |  |  |  |  |
| 8-10% | 1.01 (0.98, 1.04) | 1.01 (0.97, 1.05) | 1.03 (0.99, 1.09) | 1.03 (0.99, 1.06) |
| 11-15% | 1.02 (0.99, 1.05) | 1.05 (1.01, 1.09)* | 1.04 (1.00, 1.09) | 1.01 (0.97, 1.05) |
| 16-18% | 1.03 (0.99, 1.07) | 1.03 (0.98, 1.08) | 1.07 (1.01, 1.14)* | 1.04 (1.00, 1.09) |
| 19-25% | 1.01 (0.97, 1.05) | 1.04 (1.00, 1.09) | 1.07 (1.01, 1.12)* | 1.03 (0.99, 1.08) |
| ≥26% | 1.00 (0.95, 1.04) | 1.02 (0.97, 1.08) | 1.09 (1.03, 1.16)* | 1.05 (1.01, 1.10)* |

*p<0.05
